# Deep learning application of the discrimination of bone marrow aspiration cells in patients with myelodysplastic syndromes

**DOI:** 10.1038/s41598-022-21887-w

**Published:** 2022-11-04

**Authors:** Nuri Lee, Seri Jeong, Min-Jeong Park, Wonkeun Song

**Affiliations:** 1 Department of Laboratory Medicine, Kangnam Sacred Heart Hospital, Hallym University College of Medicine, Seoul, 07440 South Korea; 2 Department of Laboratory Medicine, Kangnam Sacred Heart Hospital, Hallym University College of Medicine, Singil-ro 1, Yeongdeungpo-gu, Seoul, 07441 Republic of Korea

**Keywords:** Cancer, Medical research

## Abstract

Myelodysplastic syndromes (MDS) are a group of hematologic neoplasms accompanied by dysplasia of the bone marrow hematopoietic cells with cytopenia. Detecting dysplasia is important in the diagnosis of MDS, but it takes considerable time and effort. Also, since the assessment of dysplasia is subjective and difficult to quantify, a more efficient tool is needed for quality control and standardization of bone marrow aspiration smear interpretation. In this study, we developed and evaluated an algorithm to automatically discriminate hematopoietic cell lineages and detect dysplastic cells in bone marrow aspiration smears using deep learning technology. Bone marrow aspiration images were acquired from 34 patients diagnosed with MDS and from 24 normal bone marrow slides. In total, 8065 cells were classified into eight categories: normal erythrocytes, normal granulocytes, normal megakaryocytes, dysplastic erythrocytes, dysplastic granulocytes, dysplastic megakaryocytes, blasts, and others. The algorithm demonstrated acceptable performance in classifying dysplastic cells, with an AUC of 0.945–0.996 and accuracy of 0.912–0.993. The algorithm developed in this study could be used as an auxiliary tool for diagnosing patients with MDS and is expected to contribute to shortening the time required for MDS bone marrow aspiration diagnosis and standardizing visual reading.

## Introduction

Myelodysplastic syndromes (MDS) are a group of hematologic neoplasms accompanied by dysplasia of the bone marrow hematopoietic cells with cytopenia. Dysplasia of MDS is divided into dyserythropoiesis, dysgranulopoiesis, and dysmegakaryocytopoiesis. Dysplasia is characterized by abnormalities in cell size, nucleation, segmentation, and granulation^1–3^. Diagnosis of MDS with single lineage dysplasia or MDS with multi-lineage dysplasia is possible only when dysplasia satisfies the criteria of 10% or more cells in each lineage. Although the detection of dysplasia plays a key role in the diagnosis of MDS^4^, it requires considerable time and effort by a hematologist for reading. In addition, since the assessment of dysplasia is subjective and difficult to quantify, a more efficient tool is needed for quality control and standardization of bone marrow aspiration smear interpretation^5^.

Recently, deep learning technology is being used to increase the accuracy of diagnoses in various medical fields. Research using artificial intelligence (AI) related to images of bone marrow specimens has mainly focused on the detection of blasts in various types of leukemia and the differentiation of normal bone marrow cells^6–11^. In the case of bone marrow aspiration specimens, research has not been conducted actively so far due to limitations of the specimen itself, including variable slides, peripheral blood dilution, and dry tap^12^. Although AI in MDS could be utilized to improve the accuracy and speed of reading and quantification of dysplastic cells, research in this field is still lacking.

In studies using deep learning for MDS so far, ‘decreased granule,’ one of the dysplasia in granulopoiesis was analyzed using the convolutional neural networks (CNN) method conducted by Mori et al.^13^. In another study, Kimura et al. developed an automated image analysis system using CNN that distinguishes MDS and aplastic anemia (AA) in peripheral blood^14^. In previous studies, only dysplasia related to decreased granules was targeted as the subject of the study. Most of the studies utilized peripheral blood or biopsy, and only a few studies used bone marrow aspirate. Further studies are needed as previous studies did not consider various cell types in bone marrow aspiration smears, which are the criteria for diagnosis. In this study, we developed and evaluated an algorithm to automatically discriminate hematopoietic cell lineages and detect dysplastic cells in bone marrow aspiration smears in patients with MDS using deep learning technology.

## Results

### Demographic and clinical characteristics

The characteristics of patients with MDS and normal slides are shown in Table 1. The median age of the patients with MDS was 71.5 and that of the normal group was 66.5. There were no statistically significant differences in the median age and sex between the two groups. Among the MDS patients, MDS with excess blast (EB)-2 accounted for 12 patients (35.5%), MDS with multi-lineage dysplasia (MLD) for 11 (32.4%), and MDS with EB-1 for 6 (17.6%). MDS with single lineage dysplasia, ringed sideroblasts-MLD, therapy-related-MDS, and MDS-unclassifiable accounted for 1, 2, 1, and 1 patient, respectively. Regarding cytogenetic characteristics, 15 patients (44.1%) had a normal karyotype, 10 (29.4%) had chromosomal gain and/or loss, and 4 (11.8%) had a complex pattern. As for the characteristics of dysplastic features, dyserythropoiesis was observed in 31 patients (91.2%), dysgranulopoiesis in 15 (44.1%), and dysmegakaryopoiesis in 21 (61.8%) (Table 2). In the case of dyserythropoiesis, nuclear budding was observed in five patients, megaloblastic change in 9 patients, and multinuclearity in 12 patients. In the case of dysgranulopoiesis, decreased granules, nuclear hyposegmentation, and unusually large size were observed in 6, 10, and 3 patients, respectively. In the case of dysmegakaryopoiesis, micromegakaryocytes were observed in 13 patients, nuclear hypolobation in 8, and multinucleation in 10.

**Table 1:** Characteristics and demographic statistics of enrolled patients.

	Enrolled patients		*P*-value
	Myelodysplasia syndromes (MDS) patients (n = 34)	Normal bone marrow group (n = 24)	
Age	71.5 (58.0–77.0)	66.5 (48.0–77.5)	0.182
Sex (male:female)	19:15	14:10	0.854
**MDS classification**		N/A	N/A
Single lineage dysplasia	1 (2.9%)		
Multi-lineage dysplasia (MLD)	11 (32.4%)		
Excess-blast-1	6 (17.6%)		
Excess-blast-2	12 (35.5%)		
Ring sideroblasts MLD	2 (5.9%)		
Unclassifiable	1 (2.9%)		
Therapy-related MLD	1 (2.9%)		
**Cytogenetics**		N/A	N/A
Normal	15 (44.1%)		
Loss and/or gain of chromosome	10 (29.4%)		
Complex pattern	4 (11.8%)		
Others	5 (14.7%)		
**Dysplastic features**		N/A	N/A
**Dyserythropoiesis**	31 (91.2%)		
Nuclear budding	5 (14.7%)		
Megaloblastic changes	9 (26.5%)		
Multinuclearity	12 (35.3%)		
Others	7 (20.6%)		
**Dysgranulopoiesis**	15 (44.1%)		
Decreased granules	6 (17.6%)		
Nuclear hyposegmentation	10 (29.4%)		
Unusually large size	3 (8.8%)		
Others	3 (8.8%)		
**Dysmegakaryopoiesis**	21 (61.8%)		
Micromegakaryocyte	13 (38.2%)		
Nuclear hypolobation	8 (23.5%)		
Mutinucleation	10 (29.4%)

**Table 2:** Number of cell images used in the study.

	Classification by algorithm			Total
	Training set	Validation set	Test set	
Total no. of cell images	6453	806	806	8065
**Cell classification**				
Normal erythrocytes (EN)	1075 (16.7%)	138 (17.1%)	119 (14.8%)	1332 (16.5%)
Normal granulocytes (GN)	2323 (36.0%)	283 (35.1%)	288 (35.7%)	2894 (35.9%)
Normal megakaryocytes (MN)	354 (5.5%)	41 (5.1%)	50 (6.2%)	445 (5.5%)
Dysplastic erythrocytes (ED)	788 (12.2%)	125 (15.5%)	100 (12.4%)	1013 (12.6%)
Dysplastic granulocytes (GD)	930 (14.4%)	103 (12.8%)	130 (16.1%)	1163 (14.4%)
Dysplastic megakaryocytes (MD)	153 (2.4%)	18 (2.2%)	19 (2.4%)	190 (2.4%)
Blasts	529 (8.2%)	54 (6.7%)	59 (7.3%)	642 (8.0%)
Others	301 (4.7%)	44 (5.5%)	41 (5.1%)	386 (4.8%)

### Detection of the cells in bone marrow aspiration slide

The total number of patch images used for the evaluation was 11,000. The manual labeling process of the nucleated cells was performed using 946 cells. A total of 756 (80%) cells were used as the training set, and 190 cells (20%) were used for validation. We achieved a Dice coefficient score of 74.7% for training and 71.1% for validation using U-net. With the same architecture, intersection over union showed 62.3% and 58.0% performance for training and validation, respectively. Using the labeling and segmentation results, the location of the cells was identified, and cell-specific cropping was conducted to obtain 555,052 cell images. We classified the 8065 cells into eight types [normal erythrocytes (EN), normal granulocytes (GN), normal megakaryocytes (MN), dysplastic erythrocytes (ED), dysplastic granulocytes (GD), dysplastic megakaryocytes (MD), blasts, and others]. The number of images for each classified cell in the dataset is listed in Table 2. We randomly divided the 8065 cells into the training (80%), validation (10%), and test (10%) sets.

### Discrimination performance of bone marrow cells with normal and dysplasia

**Figure 1:**
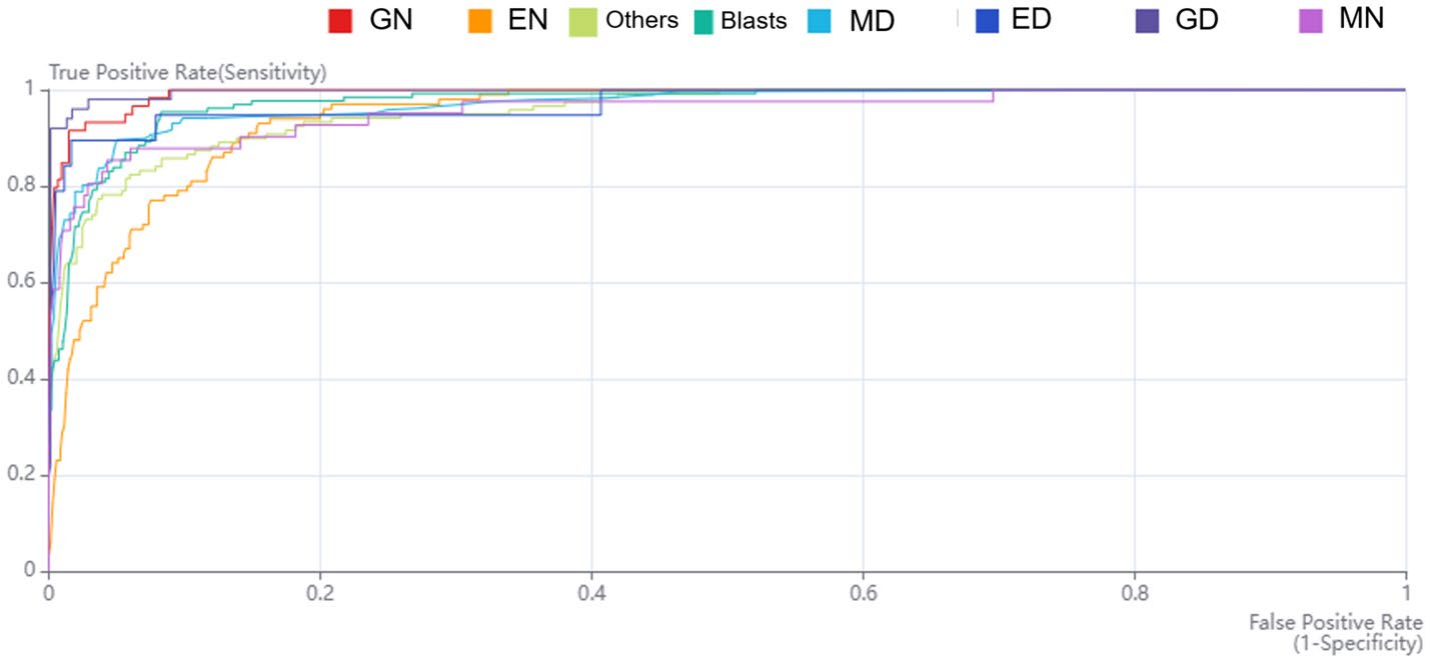
Receiver operating characteristic curves for classification of normal and abnormal cells in patients with myelodysplastic. Eight types of cells: normal erythrocytes (EN), normal granulocytes (GN), normal megakaryocytes (MN), dysplastic erythrocytes (ED), dysplastic granulocytes (GD), dysplastic megakaryocytes (MD), blasts, and others.

Table 3 presents the performance of this study, including sensitivity, specificity, accuracy, positive predictive value (PPV), negative predictive value (NPV), area under the receiver operating characteristic curve (AUC), F1 score, and average precision for the eight cell types. The AUC for GD was 0.996, with a sensitivity of 90.0% and a specificity of 99.9%. The sensitivities of ED and MD were 79.0% and 89.9%, respectively. Specificity was much higher at 99.2% for ED and 94.8% for MD. Cells with normal patterns showed decreased sensitivity and specificity compared to those with dysplastic patterns. EN, GN, and MN presented 64.0%, 79.7%, and 70.7% sensitivity and 95.0%, 99.3%, and 98.8% sensitivity, respectively. Figure 1 presents the receiver operating characteristic curve for each cell type. The figure also shows dependable results for GN (0.993), ED (0.972), MD (0.971), MN (0.955), and EN (0.945) (Table 3, Fig. 1).

**Table 3:** Summary of performance including area under the receiver operating characteristic curve for each finding in database.

	Metric							
	Sensitivity	Specificity	Area under curve	Accuracy	Positive predictive value	Negative predictive value	F1 score	Average precision
Normal erythrocytes	0.640	0.950	0.945	0.912	0.647	0.949	0.643	0.694
Normal granulocytes	0.797	0.993	0.993	0.979	0.904	0.984	0.847	0.931
Normal megakaryocytes	0.707	0.988	0.955	0.974	0.763	0.984	0.734	0.781
Dysplastic erythrocytes	0.790	0.992	0.972	0.988	0.714	0.995	0.744	0.792
Dysplastic granulocytes	0.900	0.999	0.996	0.993	0.978	0.993	0.938	0.946
Dysplastic megakaryocytes	0.899	0.948	0.971	0.931	0.906	0.944	0.902	0.956
Blasts	0.831	0.951	0.973	0.932	0.766	0.967	0.797	0.884
Others	0.782	0.956	0.954	0.931	0.756	0.962	0.769	0.845

### Analysis of true and false

The confusion matrix of the labeled cells and the prediction of cells are listed in Table 4. Among the dysplastic cells, the highest proportion of cells that the algorithm missed was normal cells of the same lineage, with 24% of ED (24/100) predicted as EN, 9.2% of GD (12/130) read as GN, and 5.3% of MD (1/19) predicted as MN. In the case of ED, 9 cells (9%) were incorrectly predicted as GD, and 7 (5.4%) were incorrectly predicted as ED in the case of GD. In the case of MD, the number of incorrectly predicted cells was one each for ED, GD, and others. Among the normal cells, megakaryopoietic cells were all read as megakaryopoietic lineage cells, and MN was predicted as MD in 5 cases (10.0%). In the case of granulopoietic cells and erythropoietic cells, 17 cases (5.9%) were incorrectly predicted as GD despite being GN, and 16 (13.4%) were predicted as ED despite EN. Supplementary Fig. 1 shows examples of correctly detected and incorrectly predicted cells using the deep learning (DL) algorithm.

**Table 4:** Confusion matrix for disease diagnosis from dataset.

Label	Prediction								
	Normal erythrocytes	Normal granulocytes	Normal megakaryocytes	Dysplastic erythrocytes	Dysplastic granulocytes	Dysplastic megakaryocytes	Blasts	Others	Total
Normal erythrocytes	93	4	0	16	2	0	3	1	119
Normal granulocytes	4	259	0	7	17	0	0	1	288
Normal megakaryocytes	0	0	45	0	0	5	0	0	50
Dysplastic erythrocytes	24	2	0	64	9	0	0	1	100
Dysplastic granulocytes	2	12	0	7	108	0	0	1	130
Dysplastic megakaryocytes	0	0	1	1	1	15	0	1	19
Blasts	0	4	0	1	2	1	47	4	59
Others	0	5	0	3	2	0	2	29	41
Total	123	286	46	99	141	21	52	38	806

## Discussion

In this study, a DL-based algorithm for dysplastic cell classification in bone marrow aspiration of MDS patients was developed and validated. This algorithm showed favorable performance when applied to the classification of dysplastic and normal cells according to the three cell lineages, including erythropoiesis, granulopoiesis, and megakaryocytopoiesis. The overall performance of AUC ranged from 0.945 to 0.996.

Research related to automated cell detection and classification in bone marrow aspiration is not easy to apply to AI because of the limitations of the sample itself, including the variability of entire slides. Also, respective AI research has not been actively conducted as compared to other fields^12^. Mori et al. analyzed a DL-based dysplasia assessment, specified for decreased granule detection system, one of the dysplastic features of MDS patients, and reported an AUC of 0.944 and an accuracy of 97.2%^13^. This study is the first cell discrimination analysis of MDS using bone marrow smear specimens. Our study expanded on previous studies to include dyserythropoiesis, dysmegakaryopoiesis, and dysgranulopoiesis. In addition, various types of dysgranulopoiesis, such as nuclear hyposegmentation and unusually large sizes other than GD, were included, and an improved algorithm could be developed. In addition, similar to the study by Mori et al., GD showed the most favorable performance among the three dysplastic cell lineages. GD showed the highest values for sensitivity, specificity, AUC, accuracy, PPV, NPV, and F1 scores. Dysgranulopoiesis accounts for the majority of nucleated cells in the bone marrow of most patients and is more specific in diagnosing MDS than dyserythropoieisis^15^. From this perspective, GD can act as a key factor in the development of a DL-applied MDS diagnosis algorithm.

In this study, specificity, AUC, and accuracy were high, but sensitivity, F1 score, and AP showed relatively low values and did not reach the reading ability of an expert. This study is a multi-classification and imbalance model; therefore, among the performance evaluation tools, the F1 score, which is defined as the harmonic mean of the precision and recall values, may be suitable for interpretation. When referring to the F1 score, the performance of this study was between 0.643 and 0.938. The gradient-weighted class activation mapping (Grad-CAM) heatmap-generating technique was applied to infer several reasons for false positives or negatives^16^. The region of interest on a bone marrow-nucleated cell in the CNN was highlighted in this technique, and the significant region of the image for prediction could be focused on, aiding the interpretation of the algorithm. Through Grad-CAM, it was found that the images correctly predicted as ED was centered on the nucleus, which is the key to the detection of dyserythropoiesis. The Grad-CAM heatmap showed that GD and MD, which were correctly predicted, and also properly detected in the nucleus and cytoplasm, and hence were suitable for dysplastic features. In contrast, in the case of ED incorrectly predicted as EN, the cytoplasm was focused instead of the nucleus. In the case of megaloblastic changes in the ED, it was difficult to read because it was predicted to be an EN. GD with decreased granules was sometimes read as EN or ED due to the hypo-granular cytoplasm, and when the hypo-granularity was severe, it was also interpreted as others. In the case of GD, nuclear hyposegmentation showed difficulty in differentiating with erythroid cells as compared to that by the pseudo-Pelger–Huet anomaly shape and/or decreased granules.

Although DL-based dysplastic cell detection has not yet shown a performance that can replace hematologists, it is important as an auxiliary tool for bone marrow-based diagnosis. Until now, most differential studies of blood cells have been conducted on peripheral blood or bone marrow biopsies^10,14,17,18^. However, in recent years, an algorithm for the differentiation of normal bone marrow cells has been developed and published, and research on dysplasia has begun, providing a basis for detecting MDS using AI^9,11,19^. Because the bone marrow aspiration slide contains many nucleated cells, and the region to be read is wide, it has several advantages when the primary classification of DL is introduced. For example, it is possible to reduce the turnaround time of test reports and count more cells, thereby increasing the accuracy of calculating the percentage of nucleated cells. In addition, instead of the diagnosis of dysplasia being made by the subjective judgment of experts, more standardization can be achieved through AI. Recently, a paper related to whole-slide image detection has been published, and it is expected that DL reading and access to digital images will increase^20^. In addition to the identification of overall normal bone marrow cells, more studies are needed to approach DL for each disease. MDS has characteristic cell morphologies and properties^4^. It is necessary to build a database that includes cell images and genomic data according to various dysplasia and develop a new approach to classify diseases and predict prognosis. In this study, the InceptionV3 architecture, a commonly used deep learning network, was utilized and may potentially be expanded for various future studies. Subsequent follow-ups to this study, such as the investigation of fully automated diagnostic approaches at the disease level for each patient and application to the pathomics of dysplastic cells are needed.

The limitation of this study is that the detailed morphological manifestations of dysplasia in each cell lineage could not be trained separately. Nevertheless, it is inferred that effective differentiation was possible by securing a sufficient number of normal cells. If dysplasia is classified according to its detailed features in the future, it is expected to achieve higher performance. Next, the ratio of the number of cells in each class cannot be unified. Granulopoiesis had a relatively large number of cells compared to the cells of other lineages; thus, there is a possibility of better performance. Therefore, in the interpretation of this study, the performance of the lineage of each cell should be determined by considering numerical differences. Finally, only cell-based performance was analyzed in this study, and additional disease diagnostic performance needs to be developed for real-world clinical application. Through follow-up research, we intend to develop an algorithm that will analyze the percentage of dysplastic cells for each lineage of all nucleated cells in bone marrow and be useful as a tool for diagnosing MDS.

In this study, we developed a classification algorithm that can distinguish between normal and dysplastic cells of three lineages in the bone marrow aspiration smear of patients with MDS. The algorithm developed in this study could be used as an auxiliary tool for diagnosing patients with MDS and is expected to contribute to shortening the time required for MDS bone marrow aspiration diagnosis and standardizing visual reading.

## Methods

### Clinical samples and whole slide scanning

**Figure 2:**
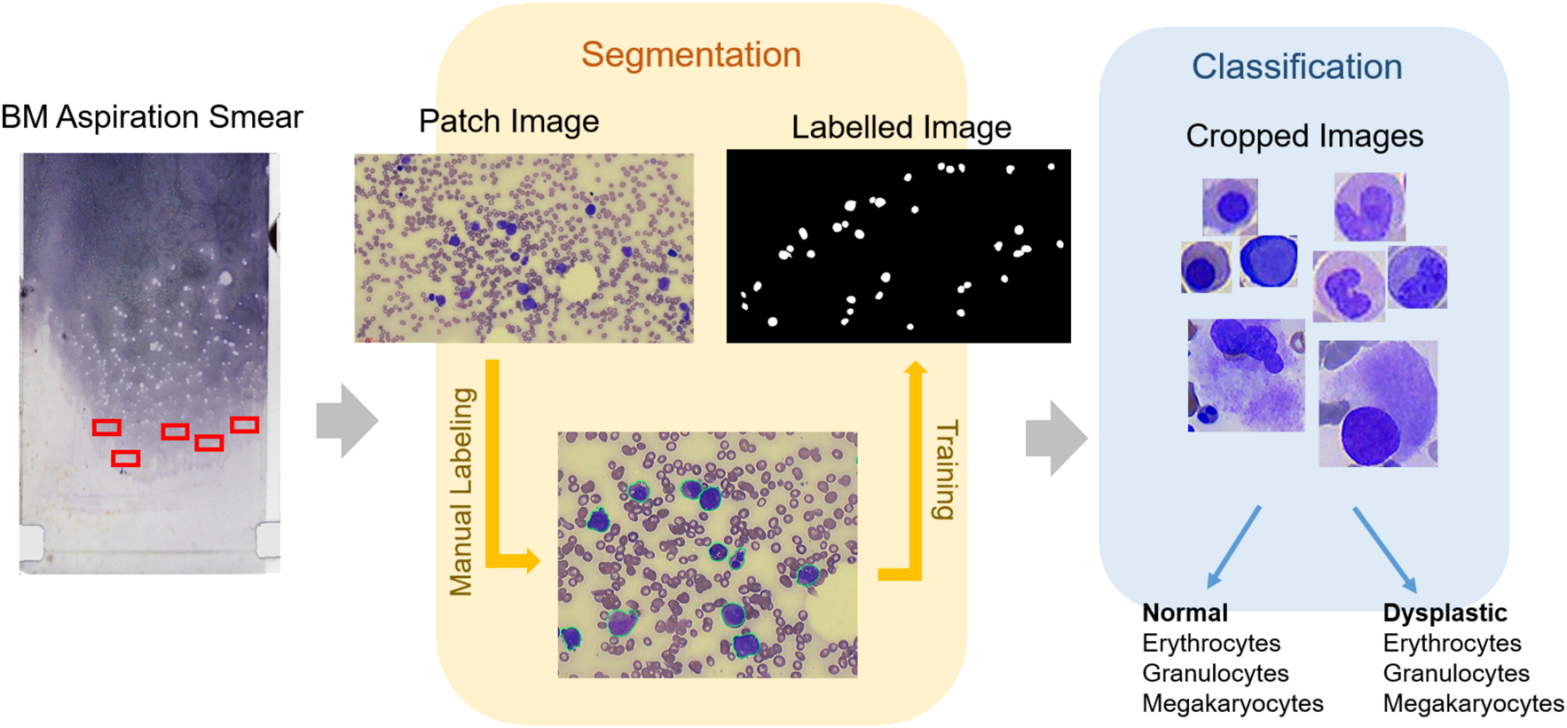
Dataset preparation and proposed framework.

The workflow of the dataset preparation and deep learning construction is presented in Fig. 2. The study was conducted according to the guidelines of the Declaration of Helsinki and approved by the Institutional Review Board (IRB) of Kangnam Sacred Heart Hospital (IRB No. HKS 2021-07-023), which waived the need for informed consent owing to the anonymized nature of the study. The data used in the study is publicly available. Bone marrow aspiration images were acquired from 34 patients diagnosed with MDS based on the WHO 2016 MDS diagnostic criteria^21^, and 24 normal bone marrow slides were required for bone marrow examination. The normal bone marrows were obtained from patients who underwent initial routine staging for lymphoma and showed no signs of hematologic malignancy and/or reactive marrow. Bone marrow smears were stained with Wright–Giemsa stain. The whole scanning of the bone marrow aspiration slide was conducted using Motic Digital Slide Assistant software version 1.0.7.61 (Motic China Group Co. Ltd., Xiamen, China).

### Automated identification of nucleated cells in bone marrow aspiration slide

**Figure 3:**
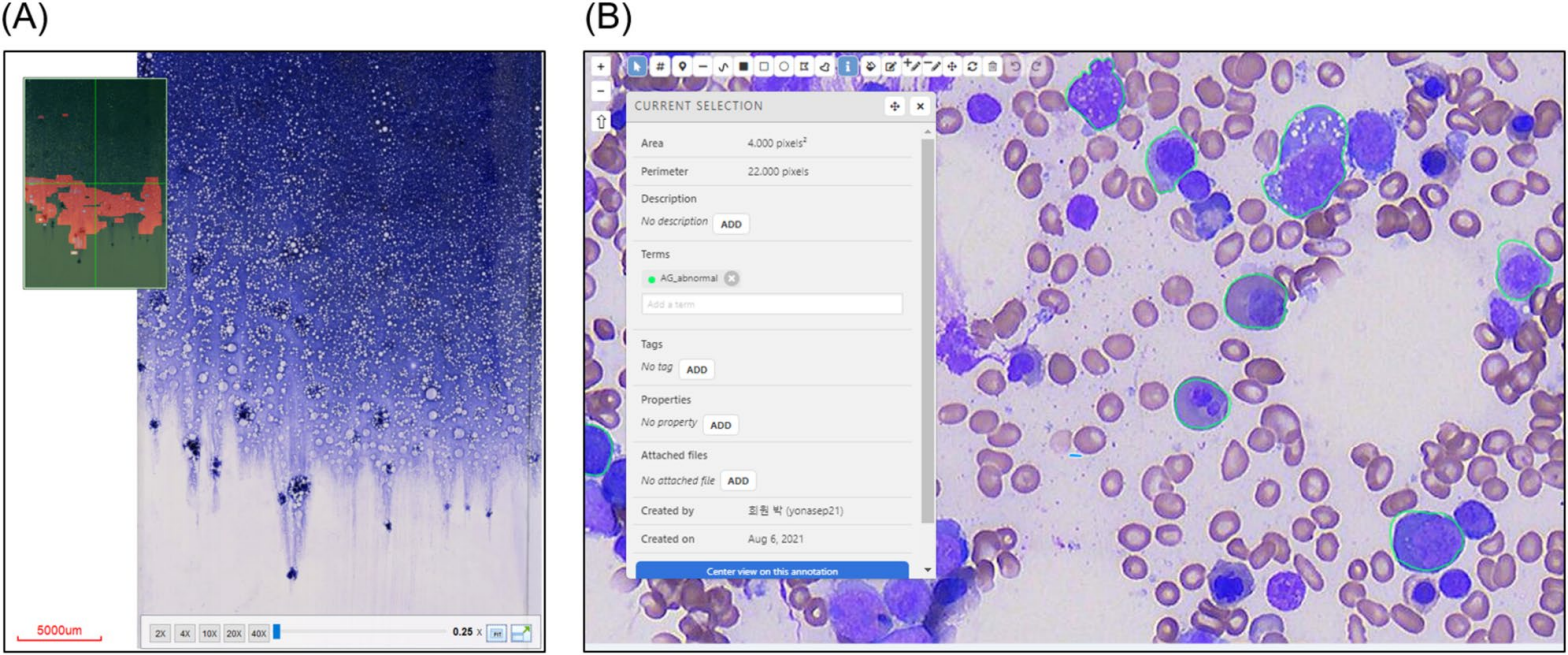
Manual labeling process of nucleated cells. (**A**) Whole slide scanning of bone marrow aspiration slide (**B**) Web-based interface for assisted annotation that enables manual labeling of nucleated cells.

Images containing the ideal zone of cell well-spread areas with nucleated cells were manually captured for the patch image. The total number of patched images was 11,000. Manual labeling of nucleated cells was performed for 946 cells and segmentation for the cell detection algorithm of patched images was developed (Fig. 3). This segmentation task, including the detection and delineation of bone marrow cells, was performed using U-Net. U-Net delineates the boundaries of nucleated cells and segments the cell area of interest from the background microenvironment.

### Development of cell identification algorithm by CNN

The cells were classified into eight types (EN, GN, MN, ED, GD, MD, blasts, and others). Normal and dysplastic cells were assigned to include and merge nucleated cells from both MDS patients and normal bone marrow specimens. All cell images were retrospectively and independently reviewed following the published standard guidelines by two hematologists with 6 and 23 years of experience in laboratory medicine, respectively. Each image was reviewed by both hematologists, and disagreements between them were resolved by consensus. We used 6453 cell images (80.0%) for training, 806 cell images (10.0%) for validation, and 806 images (10.0%) for testing. We used the metrics modules in DEEP:PHI (medical AI software: DEEPNOID, Seoul, Republic of Korea), which is an open platform that assists in DL model research. Statistical analyses were performed using the DEEP:PHI platform. We used the InceptionV3 architecture, a well-known object detection DL framework, to perform per-image classification of bone marrow cells^22^. The Grad-CAM technique was used for the interpretation and evaluation of the DL outputs^16^. An adaptive moment estimation optimizer was used for the hyperparameter settings with a learning rate of 0.0001. The batch size was 32 and the number of epochs was 200.

### Statistical and data analysis

Dice coefficient score, a statistical tool for measuring similarity, was utilized to evaluate the performance of segmentation by U-net. The AUC, sensitivity, specificity, PPV, NPV, accuracy, F1 score, and average precision were estimated in order to evaluate the performance of cell classification. The values on the curve present the degree of performance as follows: no discrimination (AUC < 0.5), acceptable (0.5 ≤ AUC < 0.7), excellent (0.7 ≤ AUC < 0.9), and outstanding (0.9 ≤ AUC)^23^.

## Supplementary Information

Supplementary Figures.

## Data Availability

All relevant database described in this study has been deposited on the Harvard Dataverse website (Lee, Nuri, 2022, “Dataset of deep learning application of the discrimination of bone marrow aspiration cells in patients with myelodysplastic syndromes,” https://doi.org/10.7910/DVN/VIRPNT, Harvard Dataverse, V1). The codes used in this study are available online (https://github.com/Nurilee822/MDS_dysplastic_classification).

## References

[1] Invernizzi R, Quaglia F, Porta MG (2015). Importance of classical morphology in the diagnosis of myelodysplastic syndrome. Mediterr. J. Hematol. Infect. Dis..

[2] Chanias I (2021). Myelodysplastic syndromes in the postgenomic era and future perspectives for precision medicine. Cancers (Basel)..

[3] Zini G (2017). Diagnostics and prognostication of myelodysplastic syndromes. Ann. Lab. Med..

[4] Kayano H (2018). Histopathology in the diagnosis of high-risk myelodysplastic syndromes. J. Clin. Exp. Hematopathol. JCEH.

[5] Goasguen JE (2016). Quality control initiative on the evaluation of the dysmegakaryopoiesis in myeloid neoplasms: Difficulties in the assessment of dysplasia. Leuk. Res..

[6] Rehman A (2018). Classification of acute lymphoblastic leukemia using deep learning. Microsc. Res. Tech..

[7] Ahmed N, Yigit A, Isik Z, Alpkocak A (2019). Identification of leukemia subtypes from microscopic images using convolutional neural network. Diagnostics (Basel, Switzerland).

[8] Pansombut T, Wikaisuksakul S, Khongkraphan K, Phon-On A (2019). Convolutional neural networks for recognition of lymphoblast cell images. Comput. Intell. Neurosci..

[9] Huang F (2020). AML, ALL, and CML classification and diagnosis based on bone marrow cell morphology combined with convolutional neural network: A STARD compliant diagnosis research. Medicine.

[10] Sirinukunwattana K (2020). Artificial intelligence-based morphological fingerprinting of megakaryocytes: A new tool for assessing disease in MPN patients. Blood Adv..

[11] Wu YY (2020). A hematologist-level deep learning algorithm (BMSNet) for assessing the morphologies of single nuclear balls in bone marrow smears: Algorithm development. JMIR Med. Inform..

[12] Elemento O (2021). Towards artificial intelligence-driven pathology assessment for hematological malignancies. Blood Cancer Discov..

[13] Mori J (2020). Assessment of dysplasia in bone marrow smear with convolutional neural network. Sci. Rep..

[14] Kimura K (2019). A novel automated image analysis system using deep convolutional neural networks can assist to differentiate MDS and AA. Sci. Rep..

[15] Goasguen JE (2018). Dyserythropoiesis in the diagnosis of the myelodysplastic syndromes and other myeloid neoplasms: Problem areas. Br. J. Haematol..

[16] Zhao J, Zhang M, Zhou Z, Chu J, Cao F (2017). Automatic detection and classification of leukocytes using convolutional neural networks. Med. Biol. Eng. Comput..

[17] Kutlu H, Avci E, Özyurt F (2020). White blood cells detection and classification based on regional convolutional neural networks. Med. Hypotheses.

[18] Brück OE (2021). Machine learning of bone marrow histopathology identifies genetic and clinical determinants in patients with MDS. Blood Cancer Discov..

[19] Jin H (2020). Developing and preliminary validating an automatic cell classification system for bone marrow smears: A pilot study. J. Med. Syst..

[20] Wang C-W (2022). Deep learning for bone marrow cell detection and classification on whole-slide images. Med. Image Anal..

[21] Arber DA (2016). The 2016 revision to the World Health Organization classification of myeloid neoplasms and acute leukemia. Blood.

[22] Szegedy, C., Vanhoucke, V., Ioffe, S., Shlens, J. & Wojna, Z. In *Proceedings of the IEEE Conference on Computer Vision and Pattern Recognition.* 2818–2826.

[23] Kim J, Hwang IC (2020). Drawing guidelines for receiver operating characteristic curve in preparation of manuscripts. J. Korean Med. Sci..

