# Supplementary material for: Deep learning application of the discrimination of bone marrow aspiration cells in patients with myelodysplastic syndromes
Source: Sci Rep. 2022 Nov 4;12:18677. doi: 10.1038/s41598-022-21887-w (PMC9636228; doi:10.1038/s41598-022-21887-w)
Supplement: Supplementary file 1 — Supplementary Figures. [file 41598_2022_21887_MOESM1_ESM.docx]

**Supplementary information**

**Supplementary Figure S1. Examples of normal and dysplastic bone marrow cells.** (A)-(H) is images for eight types of analyzed cells (normal erythrocytes (EN), normal granulocytes (GN), normal megakaryocytes (MN), dysplastic erythrocytes (ED), dysplastic granulocytes (GD), dysplastic megakaryocytes (MD), blasts, and others).

**Supplementary Figure S2. Representative images of correctly and incorrectly predicted results from from Gradient-weighted Class Activation Mapping (Grad-CAM) obtained using InceptionV3 classification model.** Examples of cases for indentification of dyserythropoiesis (A, B), and dysgranulopoiesis (C, D).

| (A) Normal erythrocytes  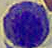 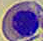 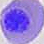 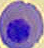 | (D) Dysplastic erythrocytes  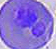 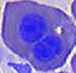 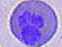 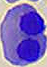 |
| --- | --- |
| (B) Normal granulocytes  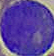 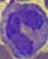 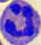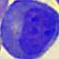 | (E) Dysplastic granulocytes  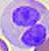 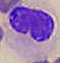 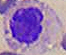 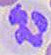 |
| (C) Normal megakaryocytes  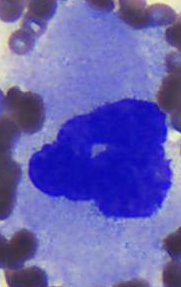 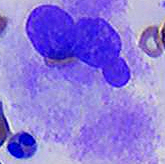 | (F) Dysplastic megakaryocytes  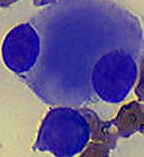 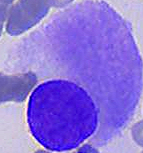 |
| (G) Blasts  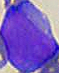 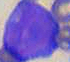 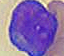 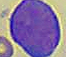 | (H) Others  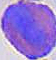 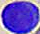 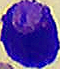 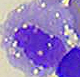 |

**Supplementary Figure S1. Examples of normal and dysplastic bone marrow cells.** (A)-(H) is images for eight types of analyzed cells (normal erythrocytes (EN), normal granulocytes (GN), normal megakaryocytes (MN), dysplastic erythrocytes (ED), dysplastic granulocytes (GD), dysplastic megakaryocytes (MD), blasts, and others).

(A) Dyserythropoiesis with correct prediction


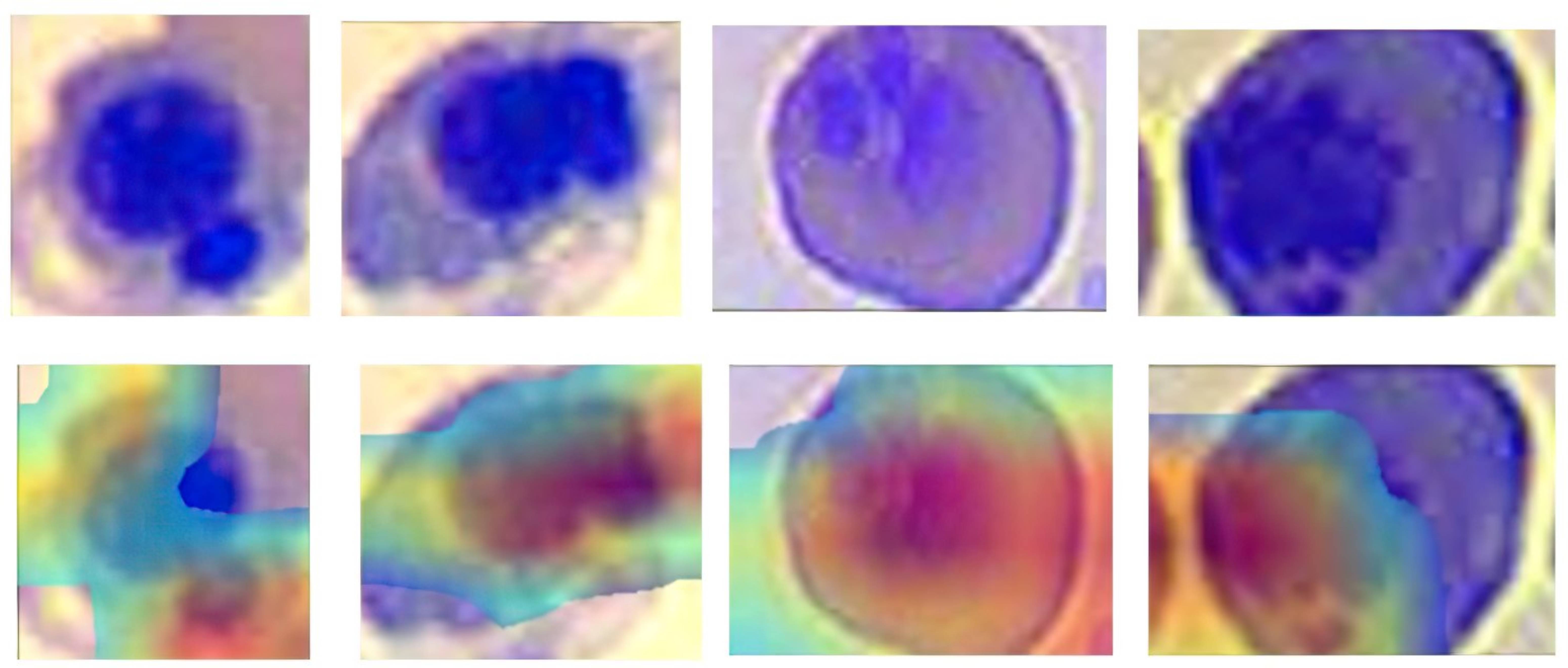


(B) Dyserythropoiesis with incorrect prediction


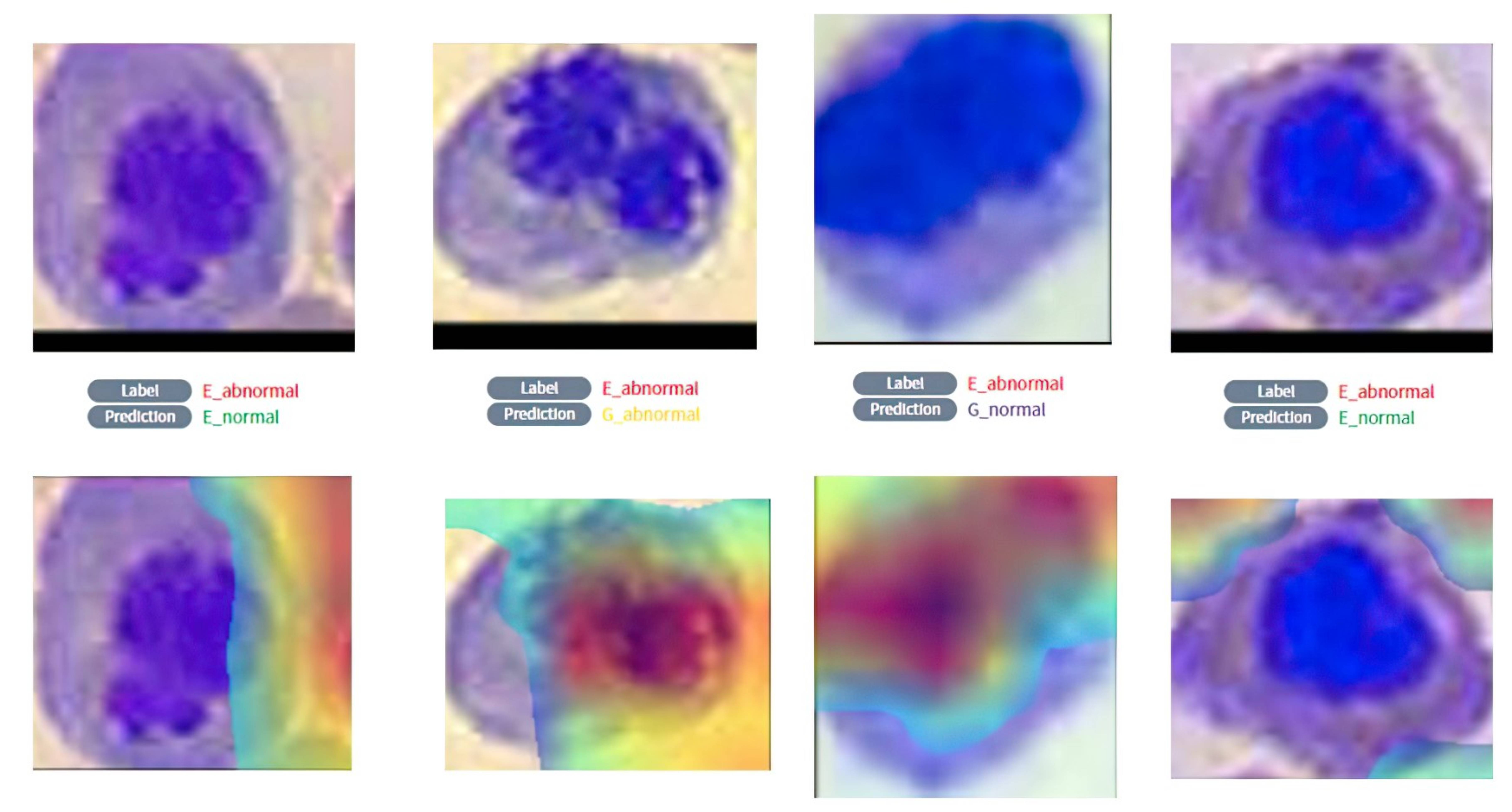


(C) Dysgranulopoiesis with correct prediction


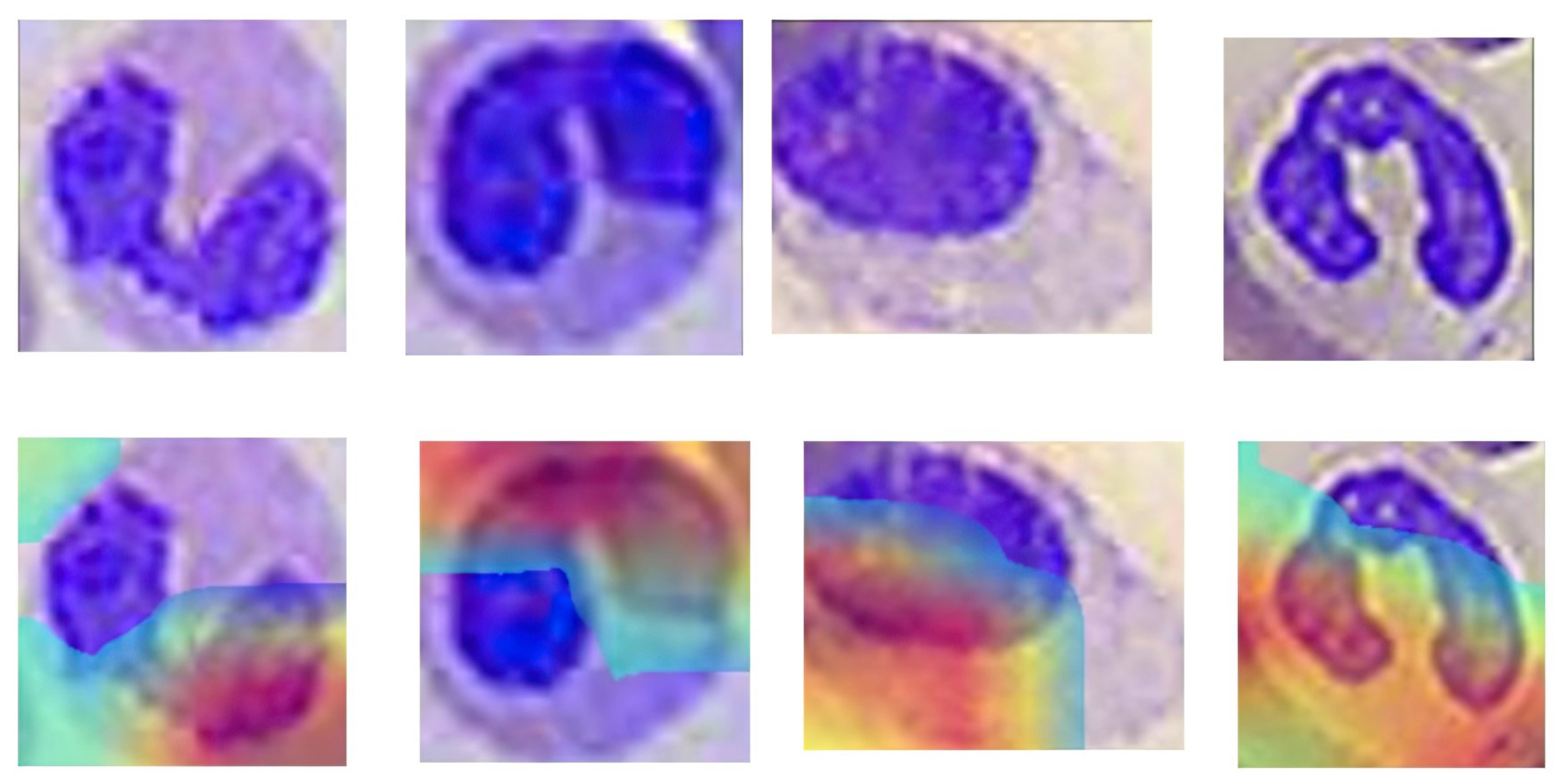


(D) Dysgranulopoiesis with incorrect prediction

**
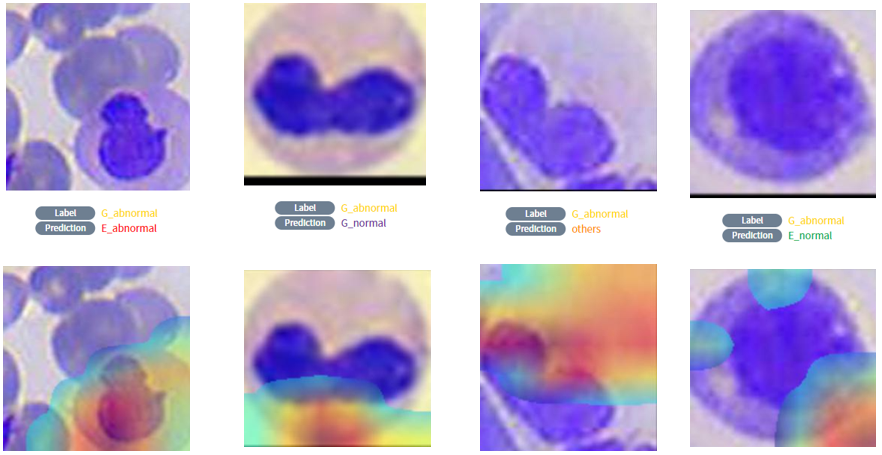
**

**Supplementary Figure S2. Representative images of correctly and incorrectly predicted results from from Gradient-weighted Class Activation Mapping (Grad-CAM) obtained using InceptionV3 classification model.** Examples of cases for indentification of dyserythropoiesis (A, B), and dysgranulopoiesis (C, D).
